# *Annual Review of Genomics and Human Genetics*: DECIPHER: Improving Genetic Diagnosis Through Dynamic Integration of Genomic and Clinical Data

**DOI:** 10.1146/annurev-genom-102822-100509

**Published:** 2023-06-07

**Authors:** Julia Foreman, Daniel Perrett, Erica Mazaika, Sarah E. Hunt, James S. Ware, Helen V. Firth

**Affiliations:** 1 European Molecular Biology Laboratory, European Bioinformatics Institute, Hinxton, United Kingdom; 2 Wellcome Sanger Institute, Hinxton, United Kingdom; 3 National Heart and Lung Institute and MRC London Institute of Medical Sciences, Imperial College London, London, United Kingdom; 4 Royal Brompton and Harefield Hospitals, Guy’s and St Thomas’ NHS Foundation Trust, London, United Kingdom; 5 East Anglian Medical Genetics Service, Cambridge University Hospitals NHS Foundation Trust, Cambridge, United Kingdom

**Keywords:** rare diseases, genetic disorders, genotype–phenotype correlation, variant interpretation, genetic diagnosis

## Abstract

DECIPHER (Database of Genomic Variation and Phenotype in Humans Using Ensembl Resources) shares candidate diagnostic variants and phenotypic data from patients with genetic disorders to facilitate research and improve the diagnosis, management, and therapy of rare diseases. The platform sits at the boundary between genomic research and the clinical community. DECIPHER aims to ensure that the most up-to-date data are made rapidly available within its interpretation interfaces to improve clinical care. Newly integrated cardiac case–control data that provide evidence of gene–disease associations and inform variant interpretation exemplify this mission. New research resources are presented in a format optimized for use by a broad range of professionals supporting the delivery of genomic medicine. The interfaces within DECIPHER integrate and contextualize variant and phenotypic data, helping to determine a robust clinico-molecular diagnosis for rare-disease patients, which combines both variant classification and clinical fit. DECIPHER supports discovery research, connecting individuals within the rare-disease community to pursue hypothesis-driven research.

## Introduction

A robust, timely diagnosis is essential for rare-disease patients, as it has major implications for patient management and treatment and substantial economic benefits for healthcare systems (35). Rare-disease research is crucial in enabling diagnosis, identifying new genes associated with Mendelian diseases, and identifying new phenotypes associated with a disorder (6). Due to the fast pace of discovery, it is essential that new research findings are made accessible to the clinical community to ensure that patient care is based on the most up-to-date knowledge.

**Figure 1:**
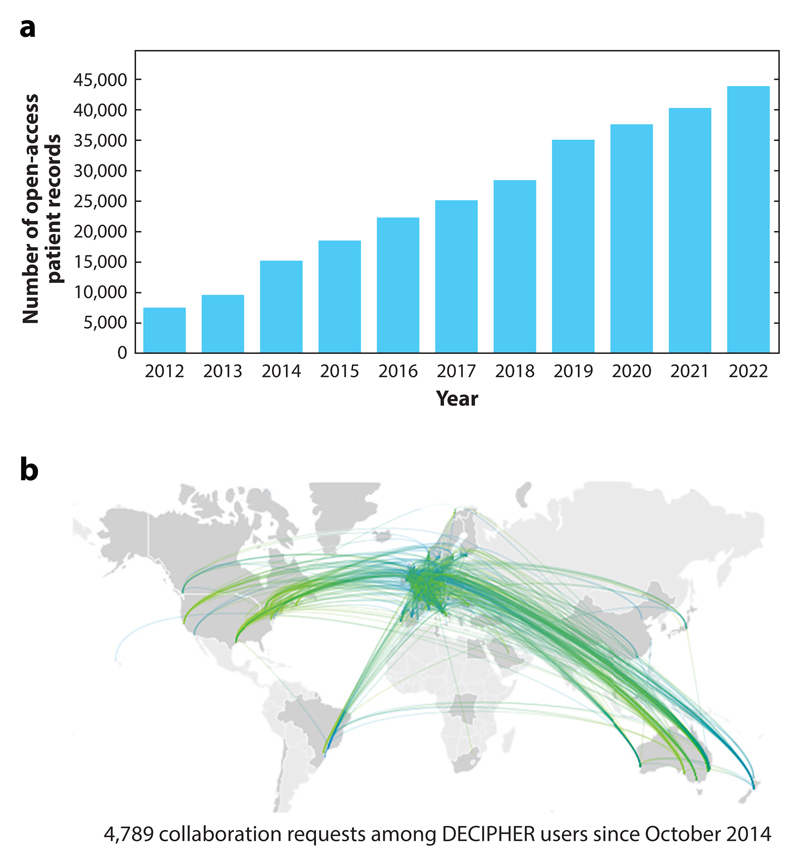
DECIPHER is a web-based platform that shares genotype and phenotype data from rare-disease patients. *(a)* DECIPHER openly shares more than 44,000 patient records. *(b)* DECIPHER enables contact between depositing centers to facilitate research and improve diagnosis. Each line on the map represents a message sent between depositing centers.

DECIPHER (Database of Genomic Variation and Phenotype in Humans Using Ensembl Resources; https://www.deciphergenomics.org) (13, 16, 29, 31, 95) is a web-based platform that shares genotype and phenotype data from rare-disease patients (Figure 1*a*). Academic genetic centers across the world deposit patient data to the platform, which shares the patient’s candidate diagnostic variant(s) and key clinical features. DECIPHER supports the deposition and sharing of patient data by clinical and research teams, who are able to obtain informed patient consent for sharing the data globally. It also supports more limited sharing between collaborating centers. The platform enables contact with the center that deposited an individual patient record to help match patients, facilitate research, and improve the diagnosis, management, and therapy of rare diseases (Figure 1*b*). DECIPHER has a broad user base that includes clinicians, clinical scientists, genetic counselors, clinical researchers, research scientists, curators, and patient support groups. The platform sits at the boundary between rare-disease research and the clinical community to ensure that the most up-to-date data are available within DECIPHER’s interpretation interfaces to enable robust clinico-molecular diagnoses. A clinico-molecular diagnosis combines both the identification of a pathogenic variant (molecular diagnosis) and the decision that the patient has the clinical symptoms of the disease in question (clinical diagnosis) (108).

A definitive clinico-molecular diagnosis is essential since it may have major implications for the patient’s prognosis, care, and treatment (108) as well as implications for other family members due to cascade screening. For some inborn errors of metabolism or inherited cancer syndromes, such interventions can be lifesaving. By contrast, errors in assigning a molecular diagnosis in an individual patient can be amplified by cascade testing, with potentially serious repercussions (1).

## Challenges in Making a Clinico-Molecular Diagnosis

### The Importance of Context

Variant classification in the absence of clinical data only occasionally allows a definitive clinical diagnosis to be made (e.g., an *FGFR3* variant in thanatophoric dysplasia). In other contexts, variant classification alone does not, in isolation, determine the clinical diagnosis. For example, most individuals carrying a truncating variant in *TTN* do not have, and will not develop, dilated cardiomyopathy (62). DECIPHER provides a platform that enables integration of the relevant phenotypic and genomic resources so that an individual patient’s data can be evaluated in the context of reference datasets, clinical datasets, and the published literature.

### Genetic and Allelic Heterogeneity

Locus heterogeneity can make it difficult to make a diagnosis since the patient’s clinical presentation may be nonspecific and give little indication of the particular genetic molecular diagnosis [e.g., intellectual disability, seizures, and congenital heart disease (107)]. Conversely, a single gene can be associated with multiple disorders caused by different variants and mechanisms of disease (allelic heterogeneity). For example, variants in *FGFR1* are known to be associated with encephalocra-niocutaneous lipomatosis (7), Hartsfield syndrome (91), hypogonadotropic hypogonadism (25), osteoglophonic dysplasia (106), and Pfeiffer syndrome (73).

### Penetrance and Expressivity

Incomplete penetrance complicates molecular diagnosis. For example, known pathogenic variants for some monoallelic inherited eye disorders are frequently observed in unaffected individuals (36). In addition, the penetrance of a disease can vary depending on the causative gene. For monogenic diabetes in clinically unselected cohorts, pathogenic variants in *GCK* display near-complete penetrance, while variants in *HNF1A* and *HNF4A* show reduced penetrance (69). Penetrance estimates vary depending on whether they are determined in a population or clinical cohort (111). Furthermore, development of manifestations over the life course is observed for many conditions [e.g., Marfan syndrome (67)]. Clinical variability in severity and organ systems affected can also impede molecular diagnosis, clouding phenotype–genotype correlation. For example, Rubinstein–Taybi syndrome ranges from the typical reported features in a continuum that overlaps with other Mendelian disorders of the epigenetic machinery (94, 99). Even patients with the same variant can display variable expressivity, as has been observed for patients with the ENST00000307340.8:c.11864G>A (ENSP00000305941.3:p.Trp3955Ter) variant in *USH2A,* with individuals exhibiting a broad range of disease manifestations in later life, ranging from good central vision to severe blindness (117). Polygenic background and genetic modifiers may play a role in suppressing or exacerbating the severity of a disease (78, 83).

### Blended Phenotypes

Blended phenotypes, when the patient has pathogenic variants in more than one gene, also confound molecular diagnosis. Two or more molecular diagnoses occur in approximately 5% of patients with a diagnosis (82). In some cases, individual phenotypic features are clearly attributable to one of the molecular diagnoses, but in others, both diagnoses may cause the same phenotypic feature(s). Different genetic conditions combined in a single patient may cause unusual and complex clinical presentations (89).

## Access to Clinical Recommendations and Guidelines

### Sequence Variants

With the rapid progress in sequencing technology and the expanding uptake, there has been a huge increase in the need for recommendations and standardization in variant interpretation. The American College of Medical Genetics and Genomics (ACMG) has published recommendations for the interpretation and reporting of results (e.g., 85). In 2015, the ACMG published joint standards and guidelines for the interpretation of sequence variants with the Association for Molecular Pathology (AMP) (86). These guidelines recommended the use of specific standard terms—pathogenic, likely pathogenic, uncertain significance, likely benign, and benign—to describe variants identified in Mendelian disorders. In addition, they described a process for classifying variants into these five categories based on criteria using typical types of variant evidence (e.g., population data and computational predictions). The strength of each evidence type was stratified into categories (supporting, moderate, strong, very strong, and stand-alone), and rules for combining criteria were used to determine the variant pathogenicity class. Global adoption of these recommendations is high. In a 2017 survey of 195 US-based and 170 international laboratories, 95% of surveyed laboratories reported using the five ACMG/AMP tiers for classifying variants in Mendelian genes, and international laboratories were just as likely to report using the guidelines as US-based laboratories (76). In addition, 97% of laboratories used approaches they considered consistent with the guidelines. A few countries have introduced their own specific guidelines; for example, in the United Kingdom, the Association for Clinical Genomic Science recommendsminor variations to these guidelines for germline variant classification for rare disease and familial cancers (26).

Since the 2015 ACMG/AMP guidelines were published, they have been adapted and refined in various ways. One significant adaptation was the translation of the guidelines into a Bayesian framework, providing a mathematical foundation, validating the approach, and providing opportunities to further refine evidence categories and combining rules (96). General clarifications and recommendations have also been published regarding the use of specific ACMG/AMP criteria, led mainly by the Clinical Genome Resource (ClinGen) Sequence Variant Interpretation Working Group (88). These include recommendations for interpreting the loss-of-function (LOF) PVS1 criterion (97), interpreting the de novo PS2 and PM6 criteria (20), and applying the functional evidence PS3 and BS3 criteria (14).

In some instances, there is a need to specify criteria based on the unique features of particular genes or diseases. For example, some rules are overly conservative in the setting of a specific disease. In the case of the BA1 criterion, the allele frequency threshold above which a variant is considered benign, the default threshold of 5% is much higher than appropriate for some diseases [e.g., *MYH7*-associated cardiomyopathies (53, 104)]. To overcome these obstacles, ClinGen variant curation expert panels (VCEPs) have been established that define the application of the guidelines for sequence variants in specific genes or diseases (88). Some of the first VCEPs’ recommendations were for *MYH7*-associated inherited cardiomyopathies (53), inborn errors of metabolism (113), *PTEN* (65), *CHD1* (57), and the RASopathies (34). There are now in excess of 50 VCEPs working to publish recommendations.

**Figure 2:**
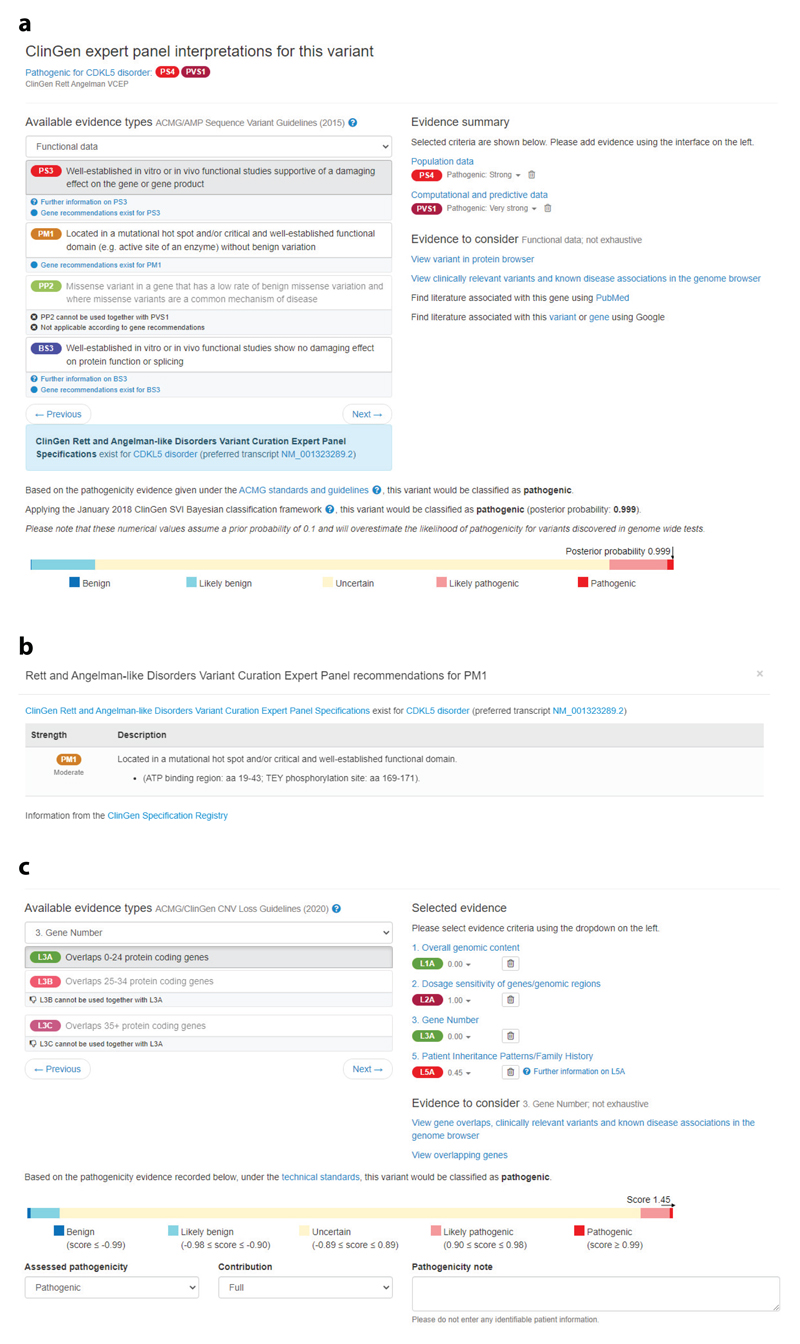
DECIPHER provides easy access to clinical recommendations for assessing variant pathogenicity. (*a*) The sequence variant interpretation interface allows users to assess pathogenicity according to ACMG/AMP guidelines. Links to ClinGen general recommendations and gene- or disease-specific recommendations are provided, in addition to ClinGen expert panel interpretations where available. (*b*) ClinGen gene- or disease-specific recommendations can be displayed in DECIPHER. The Rett and Angelman-Like Disorders VCEP example shown here has recommendations for the use of the PM1 criterion for CDKL5 disorder. (*c*) The CNV interpretation interface allows users to assess pathogenicity according to ACMG/ClinGen technical standards. Abbreviations: ACMG, American College of Medical Genetics and Genomics; AMP, Association for Molecular Pathology; ClinGen, Clinical Genome Resource; CNV, copy number variant; VCEP, variant curation expert panel.

These general guidelines and recommendations for the application of ACMG/AMP criteria, in addition to gene- or disease-specific guidelines, are continually being updated, and new recommendations are regularly published. This presents a challenge for clinical and laboratory teams to keep up to date with current guidelines and follow the latest recommendations for the particular gene and variant they are considering. DECIPHER reduces this burden by providing access to these guidelines within the sequence variant interpretation pathogenicity interface (Figure 2*a,b*). The interface is based on the 2015 ACMG/AMP framework, displaying the pathogenicity according to the 2015 ACMG/AMP combining rules in addition to the posterior probability using the Bayesian framework. Clinical teams can use their discretion to adjust the weight given to each criterion and to the outcome, particularly in the case of discrepancies in pathogenicity using the two methods. The integration of both general and gene- or disease-specific guidelines into this interface supports the harmonization of use of the criteria while providing flexibility as guidelines evolve.

### Copy Number Variants

More recently, the ACMG and ClinGen have published technical standards for the interpretation and reporting of copy number variants (CNVs) to assist clinical laboratories in the classification and reporting of CNVs (87). This quantitative, evidence-based scoring framework encourages the implementation of the five-tier classification system used in sequence variant classification. A study across nine clinical laboratories performing routine clinical CNV testing in China showed increased concordance when using these guidelines compared with the laboratory’s previous method [from 18% (41/234) to 76% (177/234) for CNVs distributed by the National Center for Clinical Laboratories] (116). To support the use of these technical standards, DECIPHER has developed a pathogenicity evidence interface that assists the annotation of pathogenicity using the CNV loss or gain guidelines (Figure 2*c*). The interface displays the different evidence types (e.g., overall genomic content and gene number) and allows the addition of relevant criteria. By default, the recommended number of points for each criterion is selected, but this can be adjusted as appropriate within the recommended ranges. To assist in the selection of only relevant criteria, contradictory criteria cannot be chosen (e.g., complete overlap of an established haploinsufficient gene or region cannot be selected in conjunction with partial overlap of an established haploinsufficient gene or region). Where additional information about the use of criteria is available, such as how to score cases for the de novo evidence criteria, this information is available within the interface. Once all relevant criteria have been selected, the final score for the CNV is displayed in addition to the calculated variant classification. Clinical teams can accept the calculated variant classification or use their discretion to select an alternative pathogenicity.

## The Importance of Accurate, Up-to-Date Genomic and Phenotypic Resources

### Reference Genome

The availability of up-to-date genomic and phenotypic resources is essential for accurate variant interpretation and making a robust clinico-molecular diagnosis. The most recent genome build, GRCh38, was released in 2013 and has been periodically updated since then (21). Compared with the previous genome build (GRCh37), GRCh38 is more contiguous and complete (95) and is more reliable for genomic analysis, producing fewer false positive structural variants (40).

In December 2020, to enable more accurate variant interpretation, DECIPHER moved from visualizing genomic data in GRCh37 to doing so in GRCh38. Deposited data were lifted over to GRCh38 when possible. For variants that have been lifted over (and variants where lift-over was attempted but not successful), DECIPHER provides an interface to compare the differences between genome builds, including the GRCh37 and GRCh38 genome browsers. Since many laboratories performing clinical sequencing continue to use GRCh37 (56), DECIPHER still supports deposition with GRCh37 coordinates, lifting over the variants to GRCh38 when possible. DECIPHER annotates variants using the Ensembl/GENCODE gene set (32), which is built by combining automated transcript annotation, using the latest evidence, with expert manual curation. To ensure that the genome build and annotation DECIPHER uses to visualize genomic variation remain current, full reannotation against the latest data is performed every time there is a new Ensembl release.

### MANE Transcripts

Displaying genomic data in GRCh38 also offers the opportunity to utilize the most recent resources being developed for variant interpretation. Reliable transcript selection is essential for accurate variant annotation; because there has been no universal standard, resources have differed in their preferred transcripts, which can confound variant interpretation. To tackle this issue, Ensembl and RefSeq (79) began a joint initiative, the Matched Annotation from NCBI and EMBL-EBI (MANE) project, to define a default set of transcripts and corresponding proteins for reporting, based on the GRCh38 assembly (71). The MANE transcript set includes both MANE Select transcripts (a single representative transcript for each human protein-coding gene) and MANE Plus Clinical transcripts (additional transcripts at loci where the Select transcript alone is not sufficient to report all currently known clinical variants). DECIPHER prioritizes MANE transcripts, assisting in the adoption of this standard.

### ClinVar, the Genome Aggregation Database, and Other Large Genomic Datasets

DECIPHER displays genomic information from many different resources that help contextualize patients’ variants to assist in interpretation. These include resources that store clinically relevant variants, such as ClinVar (55), the public version of the Human Gene Mutation Database (93), and public locus-specific databases from the Leiden Open Variation Database (30), in addition to datasets of variants that have been observed in the general population, such as the Genome Aggregation Database (gnomAD) (52) and the Gold Standard Variants in the Database of Genomic Variants (19). Many of these resources are updated regularly, and a recent paper by the Medical Genome Initiative describing best practices for the interpretation and reporting of clinical whole-genome sequencing recommended the regular updating of reference datasets (5). To ensure that DECIPHER displays the most up-to-date information, these datasets are refreshed approximately every six weeks. When a new Ensembl version is released, the variants from these resources are also reannotated (when possible) so that the most up-to-date information is always displayed.

### Gene–Disease Associations

New gene–disease associations are continually being discovered; for example, although rare variants in more than 2,000 genes have now been shown to cause developmental disorders, many more disease-causing genes remain to be discovered (23, 51). It is also important to recognize that genes reported as causative of disease and included in diagnostic tests may subsequently be found to have limited or no evidence of disease association [as happened with, e.g., genes that had been found to be associated with Brugada syndrome (43)]. This can lead to the misclassification of variants and potentially to genetic misdiagnosis. A study evaluating the clinical validity of hypertrophic cardiomyopathy (HCM) genes found that out of 33 genes frequently included in HCM testing, only 8 (24%) were classified as having definitive evidence of a gene–disease association, 3 (9%) had moderate evidence, and 22 (67%) had limited or no evidence (46). Furthermore, this study found variants in ClinVar that had been annotated as pathogenic or likely pathogenic for HCM in genes that had limited or no evidence for association with the disease. A similar picture has been reported for genes reported to be associated with dilated cardiomyopathy (49).

DECIPHER displays gene–disease association data from various high-quality resources, including the Online Mendelian Inheritance in Man (OMIM) Morbid Map (39), Gene2Phenotype (G2P) (98, 107), ClinGen (84), and the Gene Curation Coalition, a meta-resource that shares data from various resources (24). These resources are actively being curated by experts to ensure accuracy. In addition to disease associations, gene–disease validity classifications (i.e., differing levels of gene–disease clinical validity) and allelic requirements are shared.

### Mechanisms of Disease

Information on disease mechanism, as curated in the G2P project, is also displayed in DECIPHER. G2P shares mutation consequences (e.g., altered gene product structure) and variant consequences (e.g., missense variant) for each gene–disease association to assist in identifying potentially diagnostic variants and includes flags such as “restricted repertoire of mutations”— important information for a disorder such as Muenke syndrome, which is caused by a single amino acid change, Pro250Arg, in the *FGFR3* gene (72).

### Open Data Sharing

DECIPHER shares patient-level variant and phenotype data that are deposited by research and clinical teams from across the globe. Currently, more than 44,000 patient records are shared openly on the website, containing more than 57,000 variants and more than 181,000 phenotypes. The patient data shared by DECIPHER constitute a rich, live knowledge repository of variant–phenotype associations, which enables the discovery of, for example, new gene–disease and variant–disease associations. DECIPHER is a pioneering platform for data sharing and is a founding member of the Matchmaker Exchange, a Global Alliance for Genomics and Health driver project (12, 81). The Matchmaker Exchange allows the federated discovery of similar patients in databases within the network, such as GeneMatcher (40) and PhenomeCentral (80), enabling users seeking a match for patients with variants in the same gene to connect.

## Translation of Research Data Into Clinical Practice

One of the major strengths of DECIPHER is that it enables the rapid translation of research data into clinical practice, allowing new information to be accessed and used by clinical teams worldwide to improve variant interpretation.

### Gene Dosage Sensitivity Metrics

**Figure 3:**
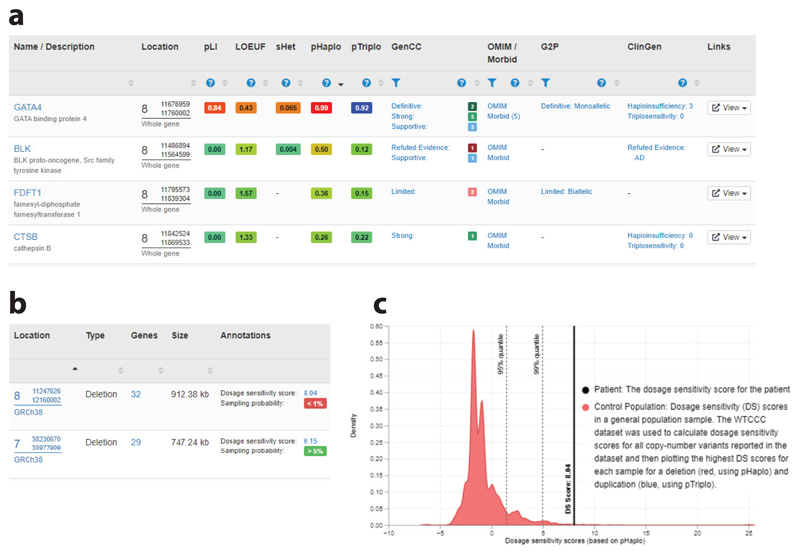
DECIPHER provides CNV annotation to assist in variant interpretation. (*a*) DECIPHER displays gene dosage sensitivity metrics in tables that list genes overlapping a patient’s CNV or structural variant to assist in the identification of candidate genes. Gene–disease associations from the OMIM Morbid Map, G2P, and ClinGen are also provided. (*b*) Dosage sensitivity scores (which are a predictor of pathogenicity) and sampling probabilities (which estimate the proportion of the general population that carry a rare deletion/duplication with a dosage sensitivity score that is as severe as or more severe than the score for the CNV being assessed) are displayed for each deposited CNV. (*c*) A graph is available for each CNV that displays the dosage sensitivity score of the CNV along with the control population. Abbreviations: ClinGen, Clinical Genome Resource; CNV, copy number variant; G2P, Gene2Phenotype; OMIM, Online Mendelian Inheritance in Man.

Detecting candidate genes when analyzing whole-exome or whole-genome sequencing is key to finding variants with possible clinical significance, and is also vital when interpreting structural variants affecting multiple genes. In addition to known gene–disease associations, predictions about genes are important because they can be used to determine the mechanisms by which variants in a gene might cause the disease and provide information about the importance of a gene when little is known about its function. Haploinsufficiency scores, such as the probability of LOF intolerance (58), LOF observed/expected upper-bound fraction (52), and predicted probability of haploinsufficiency (pHaplo) (21), indicate the tolerance of a gene to inactivation. Haploinsufficiency is known to be a common mechanism of disease, and the importance of these metrics for interpreting copy number losses is recognized in the technical standards for the interpretation and reporting of CNVs as evidence for pathogenicity (copy number loss criterion 2H). A complementary approach is to estimate the impact on fitness (e.g., survival and reproduction) for individuals who harbor heterozygous LOF variants in the gene [selection coefficient of heterozygous LOF variants (15, 102)]. DECIPHER displays these metrics on gene pages and in tables that list genes overlapping a patient’s CNV or structural variant to assist in the identification of candidate genes (Figure 3*a*). As new and improved metrics are published, DECIPHER reviews and updates the predictive scores displayed.

In contrast to haploinsufficiency, the role of triplosensitivity (duplication intolerance) in disease is less well understood, and few genes are known to be triplosensitive. In 2022, Collins et al. (21) described the generation of the probability of triplosensitivity (pTriplo) metric, which predicts the likelihood that whole-copy gain of a gene is enriched in a cohort of individuals affected by severe, early-onset diseases as compared with the general population. The pHaplo metric (described above) was also generated as part of this study. In addition to displaying these scores, DECIPHER uses these metrics to annotate CNVs with dosage sensitivity scores. Dosage sensitivity scores are based on the predicted haploinsufficiency of genes within a deletion or the triplosensitivity of genes within a duplication or other copy number gain, and indicate how likely it is that a CNV is pathogenic (Figure 3*b*). A dosage sensitivity sampling probability—an estimate of the proportion of the general population that carry a rare (observed in fewer than 1% of individuals) deletion/duplication with a dosage sensitivity score that is as severe as or more severe than the score for the CNV being assessed—is also provided (44) (Figure 3*c*). The pHaplo and pTriplo metrics and updated dosage sensitivity scores were available on the DECIPHER website less than one month after publication of the related paper, enabling these improved scores to be accessed easily.

### Splicing

Variant interpretation to date has focused mainly on the identification of pathogenic variants in the coding genome. However, there are estimates that up to 50% of monogenic disease-causing variants in some genes may affect splicing, such as in the case of neurofibromatosis type 1 *(NF1)* (4). Filtering of candidate diagnostic variants tends to focus on protein-coding consequences, and while variants in the canonical splice sites (splice acceptor or donor sites) have been annotated with deleterious consequences for some time, noncanonical splice variants have not. A 2019 study that looked at signatures of purifying selection around the splice site showed the importance of noncanonical positions, particularly the don+5 site and pyrimidine-removing mutations in the polypyrimidine region, estimating that approximately 27% of splice-disrupting pathogenic mutations within a developmental disorder cohort are in noncanonical positions (60). DECIPHER uses the Ensembl Variant Effect Predictor (VEP) (64) to annotate variants and now displays consequence predictions for noncanonical splice variants (a splice donor fifth-base variant, splice donor region variant, or splice polypyrimidine tract variant) to ensure that these are not overlooked. To further assist in splice site variant interpretation, DECIPHER provides SpliceAI annotations, which predict effects on splicing using a deep neural network (48). SpliceAI has been shown to have good sensitivity and specificity, such as in the case of *NF1* variants (37).

### Regulatory Elements

**Figure 4:**
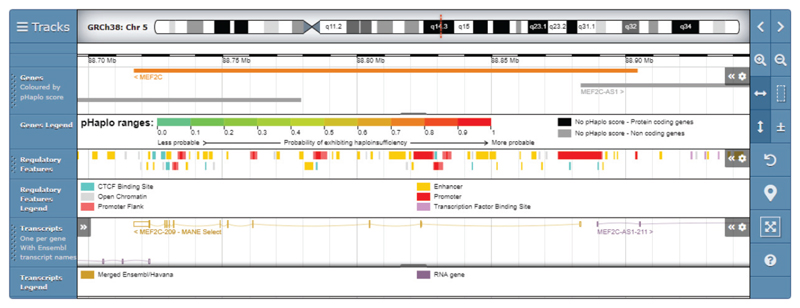
DECIPHER displays an Ensembl Regulatory Build track in the genome browser to assist interpretation of variants in the noncoding genome. The tracks displayed here are genes (colored by pHaplo score), regulatory features, and transcripts. The regulatory features available are promoters (regions at the 5’ end of genes where transcription factors and RNA polymerase bind to initiate transcription), promoter flanks (transcription factor binding regions that flank promoters), enhancers (regions that bind transcription factors and interact with promoters to stimulate transcription of distant genes), CTCF binding sites (regions that bind CTCF, the insulator protein that demarcates open and closed chromatin), transcription factor binding sites (sites that bind transcription factors, for which no other role can be determined as yet), and open chromatin (regions of spaced-out histones, making them accessible to protein interactions). The interactive Genoverse genome browser (http://genoverse.org) has been developed by the DECIPHER team. Abbreviations: CTCF, CCCTC-binding factor; pHaplo, probability of haploinsufficiency.

Variants in regulatory elements are also known to be causative of disease, such as 5’ untranslated regions in *MEF2C* that cause a developmental disorder (110). To assist interpretation of variants in the noncoding genome, DECIPHER displays an Ensembl Regulatory Build track in the genome browser (Figure 4). The Ensembl Regulatory Build incorporates data from many sources, such as chromatin marks from Roadmap Epigenomics (90), the Encyclopedia of DNA Elements (ENCODE) (27), and BLUEPRINT (2), to identify candidate regulatory elements, including enhancers, promoters, and transcription binding sites (22, 115).

### Case–Control Data

For variants associated with highly genetically heterogeneous diseases, the likelihood of interpreting a variant as pathogenic is often dependent on whether the variant has previously been identified and characterized, especially when nontruncating variants are the predominant pathogenic variant class. The interpretation of previously unseen variants is essential to provide a molecular genetic diagnosis to many patients, and for diseases that have incomplete and/or age-related onset, case–cohort data can be invaluable for interpretation. The importance of this information is reflected in the ACMG/AMP sequence variant guidelines, which include evidence for pathogenicity using case–control comparisons (the PS4 criterion, denoting that the prevalence of the variant in affected individuals is significantly higher than the prevalence in controls). As first described, this criterion is applicable only for the minority of individual variants that are recurrently observed in large case series, although modifications for the use of this criterion have been published, suggesting that it can be used less stringently in some cases (e.g., 18, 34, 53). For nontruncating variants, there is another ACMG/AMP criterion that can use information on the aggregated frequency of variants of particular classes between case and control cohorts (the PP2 criterion, denoting that there is a missense variant in a gene that has a low rate of benign missense variants and where pathogenic missense variants are common). DECIPHER is working with research and clinical teams to optimize display of case–control data and already displays these data for cardiac conditions.

### Cardiac Case–Control Data

Inherited cardiac conditions represent an important health burden, with a combined prevalence estimated at approximately 1 in 200 individuals. In 2022, DECIPHER started to share case–cohort data to support variant interpretation, aggregating variant data from cardiomyopathy and healthy volunteer (control) cohorts. Cardiomyopathies are inheritable intrinsic heart muscle diseases that are characterized by genetic heterogeneity, incomplete penetrance, age-related onset, and variable expressivity [e.g., HCM (42, 63)]. Although these are life-threatening conditions associated with a poor prognosis in some, they are medically actionable, with well-established treatments and interventions that can improve survival, reduce morbidity, and enhance quality of life (8). In families with confirmed or suspected disease, proactive clinical screening of relatives at risk, supported by genetic counseling and testing, is recommended (41). Cardiomyopathy genes also feature prominently on the ACMG secondary findings list (68), though there is no international consensus that the benefits of opportunistic screening outweigh the harms.

**Figure 5:**
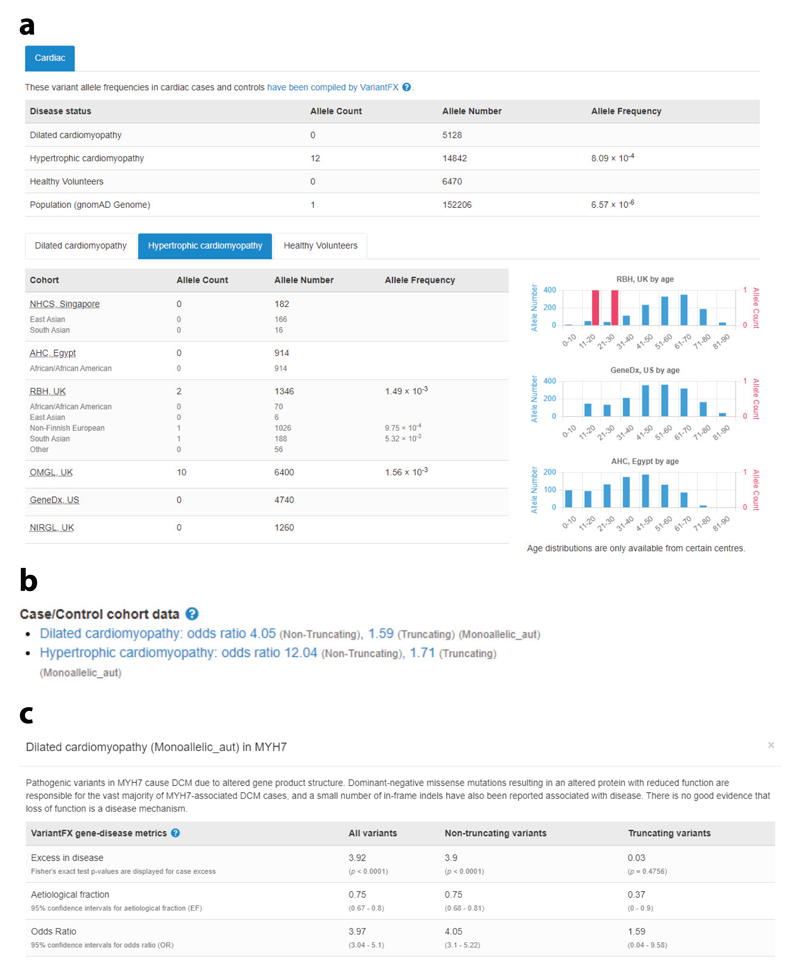
DECIPHER shares cardiac case–control data collated by Cardiac VariantFX. *(a)* For a given genomic variant, the allele frequency, allele count, and allele number observed in HCM, dilated cardiomyopathy, and healthy volunteer cohorts are displayed, assisting in the use of the PS4 criterion for variant interpretation (example displayed: https://www.deciphergenomics.org/sequence-variant/14-23429005-G-A/annotation/disease-cohorts/cardiac/hcm). (*b*) Summary cardiac case–control cohort data can be used to determine the confidence of gene–phenotype relationships and are displayed on gene tabs in DECIPHER (example displayed: https://www.deciphergenomics.org/gene/MYH7/overview/clinical-info). (*c*) Detailed case–control cohort metrics are available on a modal. Three metrics (excess in disease, etiological fraction, and odds ratio) are displayed for all variants, nontruncating variants, and truncating variants. Known mechanisms of disease are also described. In this example, distinct variants in *MYH7* lead to HCM and dilated cardiomyopathy via opposing mechanisms—that is, activating variants cause HCM, and inactivating variants cause dilated cardiomyopathy. *MYH7* is not known to be haploinsufficient, and null alleles are not associated with either condition. Abbreviation: HCM, hypertrophic cardiomyopathy.

The cardiac case–control data displayed in DECIPHER are collated by Cardiac VariantFX (45), which hosts aggregated, harmonized data from the Cardiac Variant Interpretation Consortium, a coalition of investigators in research and diagnostic laboratories seeking to understand genetic variation causing cardiovascular disease. For a given genomic variant, the DECIPHER cardiac disease cohort annotation interface displays the allele frequency, allele count, and allele number observed in HCM, dilated cardiomyopathy, and healthy volunteer cohorts (Figure 5*a*). When the variant is also in gnomAD, this information is also displayed for this population cohort. The Cardiac VariantFX data are derived from seven research and clinical centers, and the DECIPHER interface provides metrics per center. When available, ethnicity and age information, including the age of the individual in which the variant was identified, is displayed. These data are currently available for 18 genes associated with cardiomyopathies.

Case–control cohort data can also be used to determine the confidence of gene–phenotype relationships. For each gene and variant class, the frequency of rare variation in a clinical cohort (e.g., an HCM cohort) can be compared with that in a control cohort. Three metrics are reported: case excess, etiological fraction, and odds ratio. The case excess is the prevalence of rare variation in a gene in a specific disease cohort over and above the prevalence in a control cohort and estimates the proportion of cases attributable to variation in that gene. The etiological fraction is a commonly used measure in epidemiology that estimates the proportion of cases in which the exposure (in this case, a rare variant in a gene) was causal (101) and can be interpreted as an estimate of the probability that a rare variant in a particular gene is pathogenic. The odds ratio (the ratio of the odds of disease in variant carriers and noncarriers) evaluates the strength of association between variants in a gene and disease status, with a Fisher’s exact test to evaluate statistical significance. Cardiac VariantFX shares these gene-related metrics, which are displayed on gene pages in DECIPHER (Figure 5*b,c*). The odds ratio per variant class is shown for each disease where there is an excess of rare variation for that gene; the case excess and etiological fraction metrics are displayed in a modal window (pop-up), along with text describing the mechanism by which variants in that gene cause the specific disease.

The etiological fraction metric combined with clustering analysis can also be used to identify gene regions in which variants are significantly clustered in cases (100). These data are relevant for applying ACMG/AMP criterion PM1 (denoting that the variant is located in a mutational hot spot or well-studied functional domain without benign variation). Adaptations of the ACMG/AMP guidelines have been published based on this quantitative approach with the suggestion of replacing PP2 and PM1 with a single rule (PM1) with three (or more) evidence levels depending on a predefined etiological fraction for the relevant variant class. DECIPHER displays in its protein browser the location of PM1 hot spot regions for HCM- and channelopathy-associated genes, collated by Cardiac VariantFX, providing the suggested strength for applying PM1 and links to the original publications and disease-specific ACMG/AMP rule adaptations.

Throughout the DECIPHER website, information modals are available that provide descriptions of the data displayed, the data source, the retrieval date, and links to the original publications. This information is especially useful for new research datasets that may be unfamiliar to clinicians or clinical scientists.

## Integrating and Contextualizing Data

### Genomic and Protein Data

Variant interpretation involves the integration of many different evidence lines (e.g., population data, predictive data, and functional data) and subsequently many different datasets. DECIPHER’s variant interpretation interfaces bring together relevant data and contextualize a patient’s variant with respect to these datasets.

**Figure 6:**
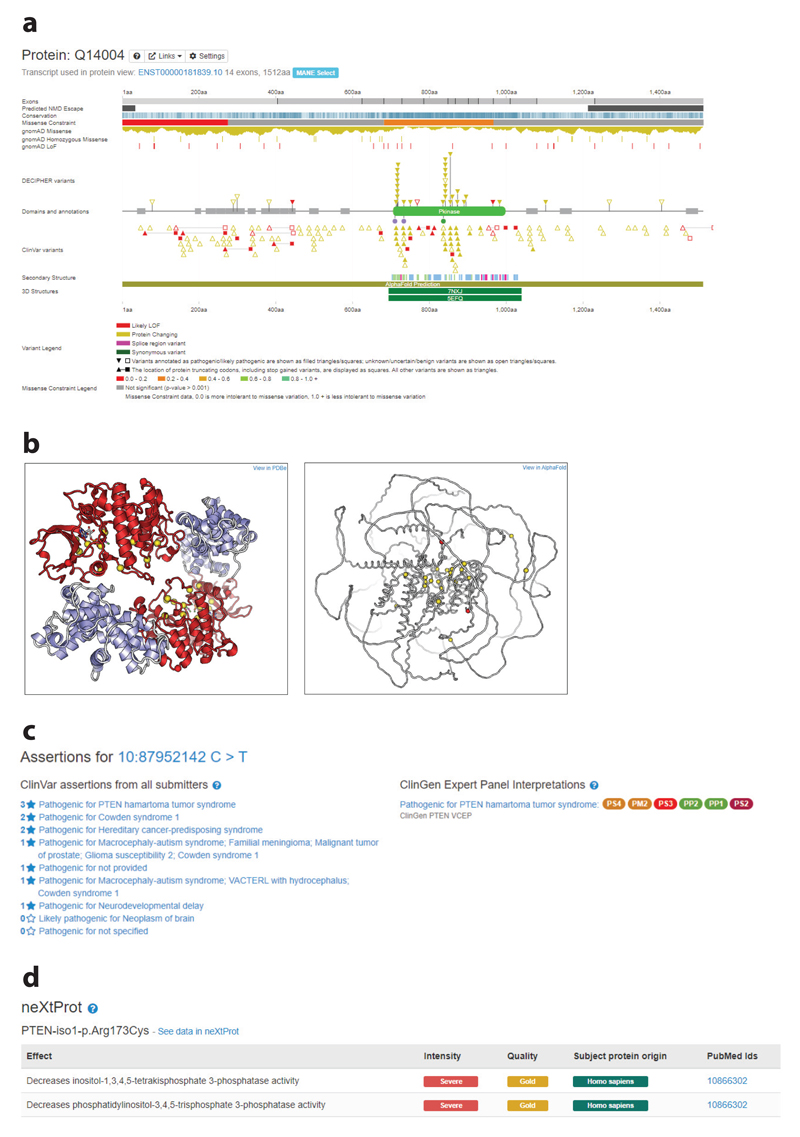
DECIPHER brings together relevant data and contextualizes a patient’s variant with respect to available datasets in addition to providing annotations. (*a*) The 2D protein browser provides a powerful genotypic overview (example displayed: https://www.deciphergenomics.org/gene/CDK13/overview/protein-genomic-info). DECIPHER and ClinVar variants are plotted individually as triangles, with filled triangles indicating a pathogenic/likely pathogenic annotation and open triangles indicating a benign/likely benign annotation or that the variant is not annotated. Due to the large number of gnomAD missense variants, these symbols are displayed as a histogram, with additional tracks displaying the location of homozygous missense, LOF, and homozygous LOF variants. Colors indicate the molecular consequence of variants predicted by Ensembl VEP; for example, red indicates a variant likely to have LOF consequences, such as a frameshift variant or splice acceptor variant, and yellow indicates a variant likely to have protein-changing consequences, such as a missense variant or in-frame deletion. (*b*) A 3D protein viewer is available that displays DECIPHER variants plotted on experimentally determined 3D structures *(left)* and 3D predicted structures *(right).* (*c*) ClinVar and ClinGen expert panel recommendations are shown (example displayed: https://www.deciphergenomics.org/sequence-variant/10-87952142-C-T/annotation/clinvar). (*d*) Functional data from the neXtProt knowledgebase are available (example displayed: https://www.deciphergenomics.org/sequence-variant/10-87952142-C-T/annotation/functional). Abbreviations: LOF, loss of function; gnomAD, Genome Aggregation Database; VEP, Variant Effect Predictor.

One of the interfaces in DECIPHER that provides a powerful genotypic overview is the 2D protein browser (Figure 6*a*). In this browser, datasets are plotted above and below a Pfam track, which provides information about protein domains and annotations (70). Clinically relevant variants from DECIPHER and ClinVar are plotted, in addition to population frequency data from gnomAD. By combining multiple datasets in a single view, the user can identify whether there are clusters of pathogenic or benign variants, as well as variants seen in gnomAD in certain regions of the protein. This information is clinically important because pathogenic variants that cause some conditions are known to cluster—for example, in the protein kinase domain for variants in *CDK13* that cause syndromic intellectual disability (38). Protein conservation, exon structure, and regional missense constraint information are also provided as separate tracks.

#### Loss-of-function variants and nonsense-mediated decay

For LOF variants, it is important to determine whether the resultant transcript is likely to undergo nonsense-mediated decay (NMD), since transcripts that escape NMD can give rise to mutant proteins that can have a potent dominant-negative activity, such as in the case of frameshift variants in *DVL1* causing Robinow syndrome (105). DECIPHER displays a predicted NMD escape track based on the 50-bp exon junction complex–dependent model and start-proximal NMD insensitivity (59, 75) to help identify variants that are likely to give rise to a transcript that escapes NMD. Since it is the location of the protein-truncating codon, rather than the location of the variant itself, that is important to predict whether the transcript is likely to undergo NMD, for likely LOF variants DECIPHER displays the location of the variant as a triangle and the protein-truncating codon as a square. In recognition of the importance of NMD escaping transcripts, recommendations for adapting the strength of the LOF ACMG/AMP criterion PVS1 include determining whether the transcript is likely to escape NMD (97).

#### Protein structures

The DECIPHER 2D protein browser also provides a track that displays secondary structure, such as helixes and turns, and a track that indicates the presence of 3D structures. Experimentally determined 3D structures from the Protein Data Bank in Europe (3) in addition to predicted structures from the AlphaFold Protein Structure Database (50) are displayed. Clicking on the structure opens an interactive 3D protein viewer (9) with all DECIPHER variants plotted (Figure 6*b*). Viewing the variants on a 3D structure is important since it may reveal, for example, that all reported variants are within a binding pocket. It has been shown that pathogenic missense variants in *ABL1* cluster in the myristoyl-binding pocket (10).

#### Annotation

DECIPHER provides annotations to assist in the interpretation of sequence variants. These annotations include gnomAD allele frequency, Ensembl VEP molecular consequence predictions (64), ClinVar/ClinGen interpretations (Figure 6*c*), functional data (Figure 6*d*), and case–cohort datasets (described above; Figure 5*a*). On the ClinVar/ClinGen tab, ClinGen VCEP assertions with respect to a disease are provided, along with the relevant ACMG/AMP criteria used to make those assertions. A modal is available that provides a summary of the interpreted evidence, the date of assertion, and a link to the relevant page in the ClinGen Evidence Repository (https://erepo.clinicalgenome.org/evrepo).

Functional annotation is recognized as evidence of pathogenicity and benignity in the ACMG/AMP guidelines (PS3/BS3), with well-established functional assays demonstrating that a variant has abnormal or normal gene or protein function. The ClinGen Sequence Variant Interpretation Working Group has also published recommendations for the application of these criteria, detailing experimental design, replication, controls, and whether the assay has been validated using human data (14). Functional data are very important for variant interpretation for novel variants and have been shown to have high sensitivity and specificity [e.g., *BRCA1* saturation genome editing (28)]. DECIPHER provides functional annotation, currently displaying data from the neXtProt knowledgebase, which provides manual annotations that capture the phenotypic effect of genetic variants (33, 112). This dataset currently includes functional annotation for variants in 130 clinically important genes, including ion channels, protein kinases, and cancer genes.

### Phenotype Data

In addition to presenting genotypic variation in its genomic and protein context, DECIPHER helps depositors share and compare patient phenotypes in order to facilitate differential diagnosis and help delineate the phenotypic spectrum of rare disorders.

#### Empirical data on frequency of phenotypes seen in a given genetic disorder

**Figure 7:**
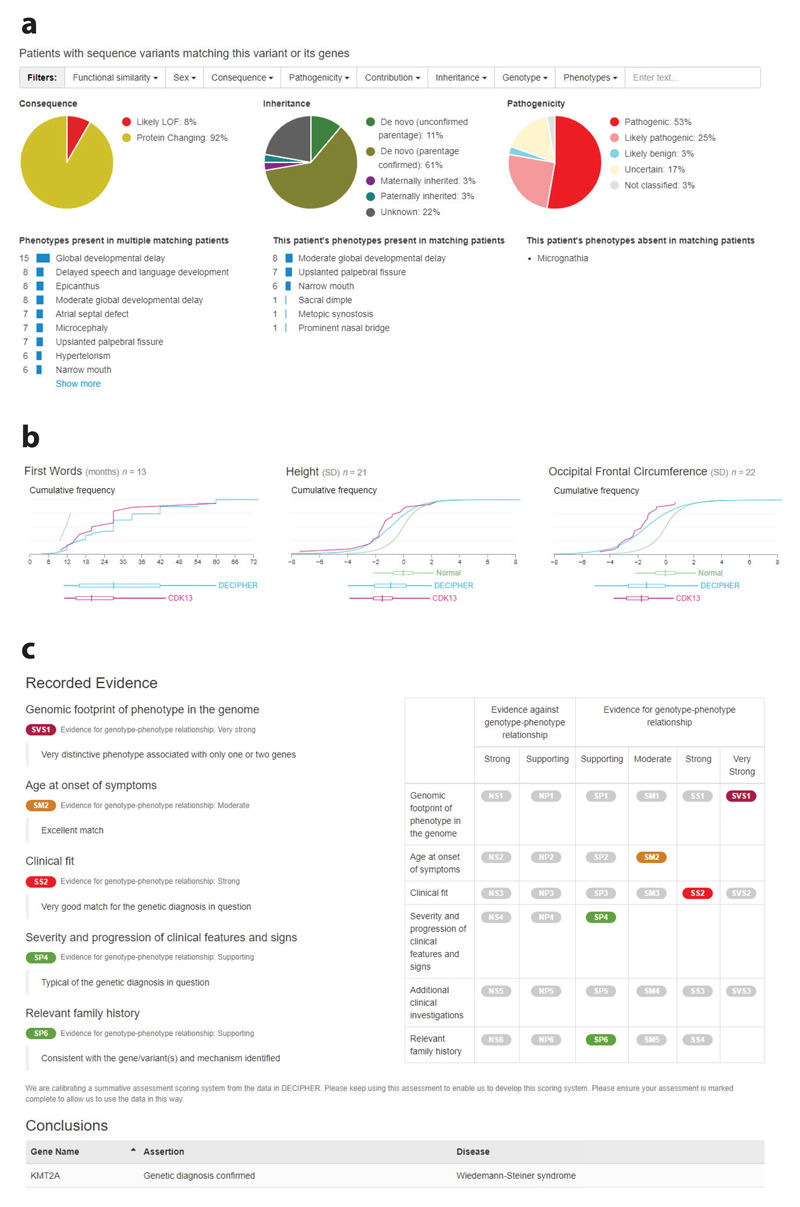
DECIPHER helps depositors share and compare patient phenotype and provides a summative assessment interface to help determine clinical fit. (*a*) The matching patient variants interface provides a summary of patients with overlapping variants. (*b*) Disorder-specific centile charts are generated from quantitative data deposited to DECIPHER (example displayed: https://www.deciphergenomics.org/gene/CDK13/overview/clinical-info). (*c*) DECIPHER provides a summative assessment interface that provides a framework to determine whether a clinico-molecular diagnosis has been established.

The matching patient variants interface provides a summary of patients with overlapping variants (Figure 7*a*). Separate interfaces are available for sequence variants within a gene, CNVs overlapping a gene, and other variants (e.g., aneuploidy or uniparental disomy) overlapping a gene. Filters are available so that the type of data being displayed can be tailored to the user’s needs, such as only displaying pathogenic/likely pathogenic variants or de novo variants. At the top of the interface summary, variant information is displayed; for sequence variants, this information comprises consequence, inheritance, and pathogenicity. Below this information, phenotype summaries are presented, such as phenotypes present in multiple matching patients. To further assist in determining the level of phenotypic overlap between a patient and what has been observed for patients with a variant in a given gene, further phenotypic summaries are also displayed. These summaries show the patient’s phenotypes that are also present in other matching patients and the phenotypes that are absent from matching patients. Individual phenotype terms [using Human Phenotype Ontology terminology (54)] are displayed rather than disease names, which allows the user to evaluate the phenotypic fit. This also allows the discovery of new phenotypes relating to a disease, disease subtypes, gene–disease associations, and variant–disease associations.

#### Disorder-specific centile charts

DECIPHER also enables the sharing of aggregated quantitative phenotype data, currently supporting developmental milestones and anthropometric measurements. For patients with a pathogenic or likely pathogenic variant in a given gene, the individual phenotype data are aggregated and displayed (Figure 7*b*). These charts can assist in determining the phenotypic similarity of a patient to other patients with pathogenic/likely pathogenic variants in a given gene. Disease-specific growth charts are also useful for the clinical follow-up of patients in terms of prognosis and monitoring of therapeutic effects (47, 74, 114).

## Integrating Genomic Variant and Phenotype to Determine a Clinico-Molecular Diagnosis

The integration and correlation of variant and clinical data are required for a clinico-molecular diagnosis. DECIPHER has a summative assessment interface that provides a framework to determine whether a clinico-molecular diagnosis has been established in a particular patient (Figure 7*c*). The framework allows the scoring of different evidence lines that demonstrate support for or against a genotype–phenotype relationship. These evidence lines include the genomic footprint of the phenotype in the genome (specifically of clinical features seen in the patient for a given gene), age at onset of symptoms, clinical fit, and severity and progression of clinical features. Information from additional clinical investigations, such as Kayser–Fleischer rings in Wilson’s disease (103) and family history, are also included. The association between a disease (OMIM gene–disease pair) and assertion is then recorded. The following assertions can be recorded: genetic diagnosis confirmed, genetic diagnosis likely, uncertain genetic diagnosis, non-penetrant (or presymptomatic) for a dominant genetic disorder, carrier of a pathogenic variant (autosomal recessive/X-linked disorder) that will never become penetrant, or no genetic diagnosis established.

The interface allows for multiple variants to be included in an assessment. This is important for recessive disorders caused by compound heterozygous variants and in cases where a combination of variants is pathogenic. Examples include *cis*-regulatory elements and common variants in *cis* that create a hypomorphic allele, such as in the case of oculocutaneous albinism (77). If the patient has a blended phenotype where more than one variant is responsible for a patient’s clinical symptoms, multiple summative assessments can be completed.

DECIPHER supports the deposition and sharing of many different variant classes, allowing CNVs and sequence variants to be included. Thrombocytopenia with absent radius (TAR) syndrome is often caused by a 1q21.1 deletion together with a sequence variant within an *RBM8A* regulatory element (11), highlighting the importance of displaying all variant types within a single interface.

## Discovery Research Using Decipher

### Catalyzing Research in Rare Disease by Global Connectivity

DECIPHER has been cited in more than 3,000 publications in the scientific literature, a testament to the importance of data sharing to drive progress in understanding rare disease. As the field advances beyond gene–disease discovery toward documentation of natural history and therapeutic trials, it will become increasingly important for patients with rare genetic disorders to be discoverable by the international research community so that they can be notified of promising research trials through their specialist physicians. Patients can then be offered the opportunity to consider whether they wish to participate. Without visibility, this opportunity is denied. Observational research and therapeutic trials need to recruit sufficient patients to document natural history and evaluate new treatments. Enabling a global reach through platforms such as DECIPHER accelerates the pace of discovery.

### An Informatics Platform for Genomic Research Studies

DECIPHER is the informatics platform used to deliver the Deciphering Developmental Disorders (DDD) study (107). DECIPHER enabled the recruitment of approximately 13,500 children with severe undiagnosed developmental disorders, with documentation of clinical features, pregnancy history, milestones, and morphometry deposited in DECIPHER by the recruiting clinical teams. The research team returned candidate variants to the recruiting clinical teams by depositing these data in the relevant DECIPHER patient record. The bespoke adaptations to DECIPHER that were built for the DDD study have now been incorporated into the main platform, so that this system is now available to other projects as either an individual study or a consortium-based study with multiple recruiting centers, and it is currently used by DDD-Africa (https://h3africa.org/index.php/ddd-africa). Since DECIPHER is free to use, it is available to support genomic medicine globally, including in low- and middle-income countries.

### Hypothesis-Driven Research in DECIPHER

**Figure 8:**
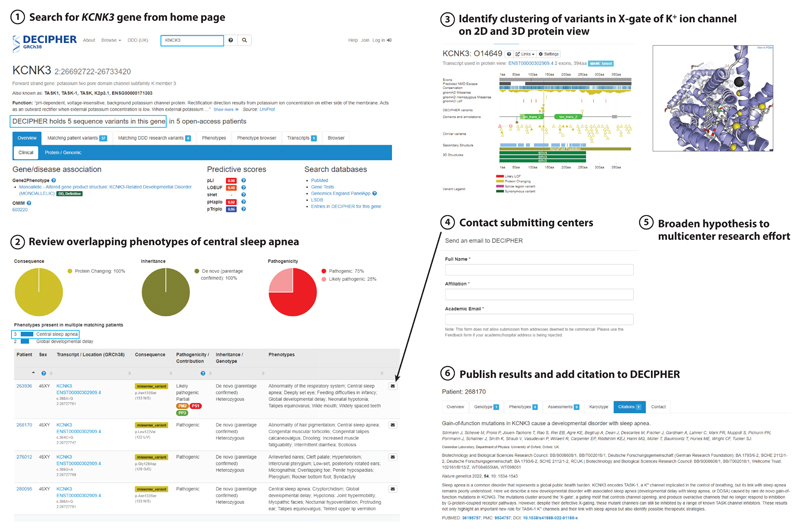
DECIPHER facilitates empirical, hypothesis-driven research, such as the identification of a novel disorder associated with *KCNK3,* which is potentially treatable by pharmacological intervention. This example comprises six steps: (①) search on the DECIPHER website for patients with variants in *KCNK3*; (②) use DECIPHER’S matching patient variants interface to view overlapping phenotypes and identify a novel phenotype (central sleep apnea) not known to be associated with *KCNK3* (③) use DECIPHER’S 2D and 3D protein viewers to observe the location of the missense variants; (④) contact the submitting centers through DECIPHER; (⑤) initiate a multicenter research effort involving clinicians, sleep specialists, genomic scientists, electrophysiologists, and pharmacologists; and (⑥) publish the results and add a citation to DECIPHER to share this information.

DECIPHER facilitates empirical, hypothesis-driven research based on candidate variants and small numbers of cases; it is complementary to big data approaches using large datasets of wholegenome sequences. Four patients recruited to the DDD study were noted to have rare de novo recurrent missense variants in the gene *KCNK3* (Figure 8, step ①), which encodes a potassium channel associated with pulmonary arterial hypertension (61). Variants associated with pulmonary arterial hypertension in this gene are hypothesized to act via a LOF mechanism. Reviewing the phenotypes (Figure 8, step ②) showed that central sleep apnea was a key clinical feature in three probands (the fourth proband was a midgestation pregnancy termination, so this phenotype could not be observed). Within the 43,854 open-access records in DECIPHER, only four other probands have central sleep apnea as a phenotype. When the variants were viewed on the 2D and 3D protein viewers in DECIPHER (Figure 8, step ③), they were seen to be tightly clustered in the ion channel domain. The tight clustering and missense nature of the de novo variants identified in patients with sleep apnea raised the possibility that these variants were causing a different disorder through a gain-of-function mechanism. In collaboration with ion channel experts, the next step (Figure 8, step ④) was to contact the submitting clinicians through DECIPHER to obtain further information and invite the clinicians and families to participate in a more detailed investigation into this potential new disorder. A multicenter collaborative investigation involving clinicians, sleep specialists, genomic scientists, electrophysiologists, and pharmacologists was initiated (Figure 8, step ⑤), resulting in the publication of a paper describing a novel disorder potentially treatable by pharmacological intervention (92) (Figure 8, step ⑥).

## Outlook and Concluding Remarks

DECIPHER enables robust clinico-molecular diagnosis by providing interpretation interfaces that contextualize patient variants with respect to up-to-date genotypic and phenotypic data. The platform integrates research data into these interfaces, enabling this valuable information to rapidly be available to the clinical community. Furthermore, DECIPHER’s pathogenicity evidence interface increases the accessibility of clinical recommendations, such as ClinGen VCEP gene- or disease-specific guidelines. Through these interfaces, DECIPHER supports ongoing reanalysis of sequencing data, which is essential due to the fast-moving pace of genomic resources. There is huge value to the ongoing iterative reanalysis of sequencing data, as actionable reclassifications can have benefits for both patients and their families. A reanalysis of a developmental disease cohort three years after the initial analysis showed that the diagnostic yield increased from 27% to 40% (109); of these new diagnoses, 69% were in new disease-associated genes, and 23% were from improved analyses (e.g., updated annotations, such as using an updated version of Ensembl VEP, and variant-filtering thresholds), highlighting the need for up-to-date analysis. Variant downgrades also have important implications; for example, a patient with a previously classified likely pathogenic *BRCA1* variant may have undergone risk-reducing surgery. Reanalysis of variants in cancer predisposition genes has shown that only a small percentage of pathogenic or likely pathogenic variants are downgraded (17, 66). Since DECIPHER is a live interface, when a variant is reclassified this information is immediately available to the rare-disease community.

DECIPHER has a broad user base that includes clinical teams, researchers, curators, and patient support groups. The bringing together of the rare-disease community, by providing a platform for all, accelerates discovery research and enables a robust clinico-molecular diagnosis for more patients.
